# Cleavage of the vaspin N-terminus releases cell-penetrating peptides that affect early stages of adipogenesis and inhibit lipolysis in mature adipocytes

**DOI:** 10.1080/21623945.2021.1910154

**Published:** 2021-04-19

**Authors:** Catherine A. Tindall, Estelle Erkner, Jan Stichel, Annette G. Beck-Sickinger, Anne Hoffmann, Juliane Weiner, John T. Heiker

**Affiliations:** a Institute of Biochemistry, Faculty of Life Sciences, Leipzig University, Leipzig, Germany; b Helmholtz Institute for Metabolic, Obesity and Vascular Research (HI-MAG) of the Helmholtz Zentrum München at the University of Leipzig and University Hospital Leipzig, Leipzig, Germany; c Medical Department III - Endocrinology, Nephrology, Rheumatology, University of Leipzig Medical Center, Leipzig, Germany

**Keywords:** Adipose tissue, internalization, obesity, proteolysis, kallikrein, serpin

## Abstract

Vaspin expression and function is related to metabolic disorders and comorbidities of obesity. In various cellular and animal models of obesity, diabetes and atherosclerosis vaspin has shown beneficial, protective and/or compensatory action. While testing proteases for inhibition by vaspin, we noticed specific cleavage within the vaspin N-terminus and sequence analysis predicted cell-penetrating activity for the released peptides. These findings raised the question whether these proteolytic peptides exhibit biological activity.

We synthesized various N-terminal vaspin peptides to investigate cell-penetrating activity and analyse uptake mechanisms. Focusing on adipocytes, we performed microarray analysis and functional assays to elucidate biological activities of the vaspin–derived peptide, which is released by KLK7 cleavage (vaspin residues 21-30; VaspinN). Our study provides first evidence that proteolytic processing of the vaspin N-terminus releases cell-penetrating and bioactive peptides with effects on adipocyte biology. The VaspinN peptide increased preadipocyte proliferation, interfered with clonal expansion during the early stage of adipogenesis and blunted adrenergic cAMP-signalling, downstream lipolysis as well as insulin signalling in mature adipocytes.

Protease-mediated release of functional N-terminal peptides presents an additional facet of vaspin action. Future studies will address the mechanisms underlying the biological activities and clarify, if vaspin-derived peptides may have potential as therapeutic agents for the treatment of metabolic diseases.

## Introduction

Serine protease inhibitors (serpins) represent the largest family of protease inhibitors and are present in all domains of life – bacteria, archaea, eukaryotes, and also in viruses [1]. All serpins exhibit a highly conserved structure of nine α-helices and three β-sheets even though sequence homology is rather low [2]. Their inhibition mechanism relies on a protruded reactive centre loop (RCL) on top of the molecule, which serves as bait for the target protease(s) [3]. When these recognize and cleave a specific RCL sequence, the hydrolysis reaction is halted after formation of the covalent protease-substrate complex due to rapid insertion of the cleaved RCL into the central β-sheet. In the process, the protease is translocated to the bottom of the serpin molecule. In this position the active site is dysfunctional and the inactive protease remains trapped in a permanent complex with the inhibitor [4].

Our recent work focused on the visceral adipose tissue-derived serpin vaspin (SERPINA12). Vaspin is a multifaceted serpin with many different biological functions in various cell types and tissues [5] and vaspin expression and function is associated with various metabolic disorders and comorbidities of obesity such as type–2 diabetes [6] and atherosclerosis [7]. Vaspin overexpression and administration improved glucose tolerance and enhanced insulin action in vivo [8–11]. Two protease targets of vaspin have been identified so far, i.e. kallikrein-related peptidases 7 and 14 (KLK7 and KLK14) [9,12] and experiments using non-inhibitory vaspin variants have linked vaspins inhibitory activity to KLK7-mediated insulin degradation [9,13]. Vaspin also reduces cytokine-induced inflammation in adipocytes [14] and transgenic overexpression prevented adipose tissue inflammation in obese mice, resulting in improved systemic insulin sensitivity [8]. Complementing these findings, adipocyte-specific knockout of KLK7 also prevented obesity-induced inflammation, preserved insulin sensitivity and reduced weight gain in obese mice [15].

Both vaspin target proteases are inhibited by the classical serpin substrate-suicide mechanism after cleavage of the reactive centre loop after Met^378^ [9,16]. In addition to cleavage within the RCL, both KLK proteases rapidly cleave the vaspin N-terminus, i.e. after Tyr^30^ for KLK7 [16] and after Arg^28^ for KLK14 [12]. Lack of these N-terminal amino acids does not affect vaspin function as a protease inhibitor nor the rate-accelerating effect of heparin binding [16,17].

N-terminal cleavage is known from other serpins. The most prominent example is the non-inhibitory angiotensinogen (SERPINA8) as essential part of the renin-angiotensin-system. N-terminal cleavage by the aspartyl-protease renin releases the decapeptide angiotensin I, which is subsequently converted to angiotensin II by angiotensin converting enzyme to mediate vasoconstriction and increase blood pressure [18]. A second example is protein C inhibitor (PCI, SERPINA5). This inhibitory serpin was found to be internalized by neutrophils [19] and later the N-terminal A+-helix sequence was identified as the protein transduction domain (PTDs) enabling internalization of PCI into cells [20]. N-terminal processing by the membrane-anchored protease testisin cleaves this basic sequence, releasing the PTD and thus functioning as a regulatory mechanism for PCI internalization [21].

The vaspin-derived N-terminal peptide(s) are predicted to have cell penetrating activity, but biological activity and physiologic functions of these peptides have not been investigated so far. The current study had two objectives. We synthesized various N-terminal vaspin peptides to investigate whether these indeed are cell-penetrating peptides (CPP) and analysed contributing uptake mechanisms. Second, by performing microarray analysis and functional assays, we elucidated biological activity of the released vaspin peptide in 3T3-L1 and primary mouse adipocytes.

## Methods

### Materials, proteins, peptide synthesis, and analytics

Chloroquine, chlorpromazine, CL316,243, cytochalasin D, Ed64, forskolin, heparin (average molecular weight of 18 kDa), insulin, leupeptin, MG132, pepstatin A and poly-D lysine were from Sigma-Aldrich (St. Louis, MO, USA). Recombinant human vaspin wild type was expressed in *E. coli* and purified as described previously [16]. Recombinant vaspin was incubated with different proteases (KLK1, KLK4, KLK7, KLK14, MMP9 and factor Xa; all from R&D Systems, MN, USA) as previously described and according to manufacturer’s instructions [9,12]. Fluorescence-labelled 5(6)-carboxytetramethylrhodamin-HIV–1 TAT protein (47-57; TAMRA-YGRKKRRQRRR-NH₂) was from Bachem (H-7524; Bachem, Bubendorf BL, Switzerland). Peptides were synthesized by automated solid-phase peptide synthesis on an automated multiple peptide synthesis robot system (Syro, MultiSynTech, Witten, Germany) using Fmoc/tBu strategy and were purified and analysed as previously described [22]. N-terminal TAMRA-labelling was done before cleavage from the resin [23]. Purity of all peptides was ≥90%. Columns, flow rates and gradients are listed in Supplementary Table S1. For mass spectrometry (MS), peptides and proteins were concentrated and desalted using ZipTip C18-filter tips (Merck Millipore, Burlington, MA, USA) and analysed using super-2,5-dihydroxybenzoic acid matrix by MALDI-TOF MS on an Ultraflex III mass spectrometer (Bruker, Billerica, USA).

### Prediction of cell-penetrating activity

Sequence analysis for cell penetrating activity was done using the CPPred-RF server (http://server.malab.cn/CPPred-RF/ [24],).

### Circular dichroism (CD) spectroscopy

Circular Dichroism spectra were acquired on a J-1500 CD spectrophotometer (Jasco, Cremella, Italy) equipped with a Peltier-type temperature control system at 25°C as previously described [16]. All spectra were baseline-corrected by subtraction of the buffer spectrum. Peptide concentrations were 50 µM in 10 mM phosphate buffer (pH = 7.2) with or without the addition of 50% TFE to stabilize α-helical structures [25]. Measured ellipticity Θ was converted into mean residue molar ellipticity [Θ] in deg*cm^2^*dmol^−1^ as described previously [26].

### Cell culture and experiments

Subconfluent 3T3-L1, primary subcutaneous and epididymal adipocytes (scAT and epiAT, from male C57BL/6NTac mice), immortalized brown adipocytes (imBA) [27], HEK293 and HepG2 cells were cultured in DMEM supplemented with 10% FCS in a humidified atmosphere containing 5% CO_2_ at 37°C. Immortalized and primary adipocytes were differentiated into mature adipocytes as previously described [14,15]. To investigate the mechanism of peptide uptake, specific inhibitors and modulators of endocytic pathways were used. 3T3-L1 preadipocytes were starved with serum-free DMEM for 30 min and subsequently incubated with TAMRA-labelled peptides, with or without inhibitors at 37°C. Chlorpromazine (inhibitor of clathrin-coated pit formation, at a concentration of 15 µM), cytochalasin D (inhibitor of actin-depolymerization, at 5 µM), chloroquine (inhibitor of endosomal acidification, at 100 µM), Ed64 (cysteine protease inhibitor, at 10 µM – 50 µM) and leupeptin (cysteine, serine and threonine protease inhibitor, at 50 µM – 500 µM) were used for 30 min, pepstatin A (aspartyl protease inhibitor, at 1 µM – 10 µM) and MG132 (proteasome inhibitor, at 1 µM – 10 µM) for 2 h prior to peptide addition. Heparin was added to the peptide solution at 500 µg/ml prior to cell treatment. Peptide uptake was then analysed by fluorescence microscopy or high content imaging.

For signal transduction assays, fully differentiated 3T3-L1 adipocytes were treated with or without 1 nM, 10 nM and 100 nM of VaspinN for 24 h, followed by insulin (100 nM for 15 min), β-adrenergic agonist CL316,243 (1 µM for 15 min), or adenylyl cyclase agonist forskolin (1 µM for 15 min) respectively, prior to Western Blot analysis. For microarray analysis to determine the effect of VaspinN on gene expression in adipocytes, fully differentiated primary adipocytes from subcutaneous adipose tissue were treated with 100 nM of VaspinN for 24 h before harvesting, RNA isolation and microarray analysis. Solvent (water) treated cells served as controls.

Experiments to isolate primary cells from animals were approved by the local authorities of the state of Saxony (Germany), as recommended by the responsible local animal ethics review board (Landesdirektion Leipzig, Germany, T02/19).

### Fluorescence microscopy

All measurements of peptide uptake were performed in living, non-fixed cells grown in eight well plates (ibidi GmbH, Gräfelfing, Germany). On the day before the experiment, cells were seeded at a density of 30.000/well (3T3-L1, imBA, primary scAT and epiAT), 150.000/well (HEK293) or 40.000/well (HepG2) and cultured in DMEM supplemented with 10% FCS. For HepG2 cells, plates were additionally coated with poly-D-lysine (0.01% in PBS) for 20 min, followed by two washing steps with PBS, in order to achieve a uniform growth. Cells were serum-starved for 30 min followed by incubation with the peptides (30 min at 37°C). Nuclei were stained with Hoechst 33342 (2 µg/ml). After incubation, the peptide solution was removed and cells were washed with acidic buffer (50 mM glycine, 100 mM NaCl, pH = 3) and covered with DMEM again. Intracellular peptide uptake was analysed immediately using an Axio Observer microscope with an ApoTome Imaging System (Carl Zeiss, Oberkochen, Germany).

### High content imaging

To quantify intracellular fluorescence of TAMRA-labelled peptides, 12.000 cells/well of 3T3-L1 or 50.000 cells/well of HEK293 were seeded in 96-well plates (Greiner µclear black, 655090) and cultivated for 24 h at 37°C and 5% CO_2_. For 3T3-L1 cells, wells were coated with 0.2% for 2 h at 37°C prior to seeding. Cell treatment was performed as described above. For each peptide concentration, at least three technical replicates (wells, single site per well) were measured using the ImageXpress Micro Confocal High-Content Imaging System 2018 with MetaXpress software version 6.5.3.427 (Molecular Devices, San José, USA). Measurements were performed using a 40x S fluor objective (unbinned images) with the two excitation wavelengths, TRITC (555/28 nm) and Hoechst 33342 (377/54 nm). In the MetaXpress software, laser-based autofocusing was enabled and was used to set the instrument to the plate bottom. Autofocus options were set to laser with z-offset. TRITC fluorescence was set to 3000 ms and Hoechst 33342 to 1000 ms exposure time and FL shading only correction was used. During measurements, the internal conditions were set to 37°C and 5% CO_2_. For analysis, granule integrated intensity per cell was evaluated using standard algorithm. TRITC granule integrated intensity was determined with granule size between 1 µm – 10 µm and 250 grey levels intensity above local background, whereas nuclei were defined as 5 µm – 30 µm and evaluated with 40 grey levels intensity above local background. Experiments were repeated at least twice.

### RNA preparation, quantitative real-time-PCR (qPCR), and microarray analysis

RNA isolation from cultured cells was done using RNeasy Lipid Tissue Mini kit (Qiagen, Hilden, Germany) as specified by the manufacturer. qPCR was performed using the LightCycler System LC480 and LightCycler-DNA Master SYBR Green I Kit (Roche, Mannheim, Germany). Adipocyte gene expression was calculated by ΔΔCT method and normalized to *NoNo* levels in each sample [28]. Primer sequences are listed in (Supplementary Table S2). Before microarray analysis, RNA integrity and concentration were examined on an Agilent Fragment Analyser (Agilent Technologies, Palo Alto, CA, USA) using the HS RNA Kit (Agilent Technologies) according to the manufacturer’s instructions. Microarray analysis was conducted at the Core Unit DNA Technologies (Core Facilities of the Faculty of Medicine; University of Leipzig). cRNA was prepared from 100 ng of total RNA and hybridized to GeneChip Clariom S arrays (Thermo Fisher Scientific) according to the manufacturer’s instructions. The arrays were scanned with a third-generation Affymetrix GeneChipScanner 3000. GeneChip data were extracted from fluorescence intensities and were scaled in order to normalize data for inter-array comparison using Transcriptome Analysis Console (TAC) 4.0.2 software according to the instruction of the manufacturer (Thermo Fisher Scientific). CEL files (raw data) were preprocessed according to the oligo Bioconductor R package (v1.50.0 [29],) to perform a deconvolution method for background correction, quantile normalization using the Robust Multichip Average (RMA) algorithm [30] for summarization. The Biobase (v2.46 [31],) and oligo R Bioconductor packages was used for the quality control of the raw and normalized data. No outliers were detected performing the outlier detection test of the arrayQualityMetrics (v3.42 [32],) Bioconductor R package. Control samples were removed and genes expressed below the threshold were filtered from the normalized data based on their transcript median intensities. Therefore, transcripts that do not have intensities larger than a threshold of 4 in at least as many arrays as the smallest experimental group are excluded [33]. The differentially expressed genes (DEGs) between control and VaspinN-treated primary subcutaneous adipocytes (scATCon and scATPep, respectively) were screened using the Linear Models for Microarray data (LIMMA) method implemented in the limma (v3.42 [34],) statistical R package. Array weights are considered during the analysis of differentially expressed genes (DEGs) to increase the signal-to-noise ratios. The threshold for identification of DEGs was set as *p-*value <0.01 and |log_2_ fold change (FC)| ≥0.5. A broad gene list enrichment analysis for pathways and GO terms was performed using Enrichr (www.amppharm.mssm.edu/Enrichr [35,36];) and all DEGs (*p-*value <0.01) irrespective of fold change. DEGs are listed in Supplementary Table S3. Microarray data have been deposited in the ArrayExpress database at EMBL-EBI (www.ebi.ac.uk/arrayexpress [37],) under accession number E-MTAB-9592.

### Cell viability, preadipocyte proliferation, clonal expansion and adipogenesis

Membrane toxicity was measured using the Cytotoxocity Detection kit (11644793001, Roche, Basel, Switzerland). 3T3-L1 preadipocytes were seeded in 24 well plates (4*10^4^ cells/well) and cultivated for 24 h or differentiated as described above. Cells were incubated with different VaspinN peptide concentrations for 1 h or 24 h at 37°C. Supernatants of each well were diluted 1:2 (3T3-L1 preadipocytes) or 1:50 (differentiated 3T3-L1 adipocytes), transferred in 96-well plates. Reaction mix was added and incubated for 30 min. Membrane toxicity was determined by measuring the absorbance (490 nm; 620 nm as reference) using a microplate reader (Tecan, Männedorf, Switzerland). To evaluate VaspinN peptide effects on cell proliferation and viability, WST-1 cell proliferation kit was used following the manufacturer’s protocol (11644807001, Roche). Cells were seeded in 96-well plates at 2.5 × 10^3^ cells/well in the presence of different concentrations of VaspinN peptide (0 nM, 1 nM, 10 nM and 100 nM). After 24 h, WST 1 reagent was added and incubated for another 3 h at 37°C and 5% CO_2_. Absorbance (450 nm) was measured using a microplate reader (Tecan). Effects of the VaspinN peptide on mitotic clonal expansion (MCE) were analysed using bromodeoxyuridine (BrdU) as previously described [38], with minor changes. Cells were treated with VaspinN peptide (1 nM, 100 nM) either chronically (added to culture medium every day beginning with confluency at day 0) or acutely (only added to induction medium at day 2). BrdU was then added to cells 20 h post induction and DNA synthesis was quantified 4 h later, i.e. 24 h post induction of adipogenic differentiation (day 3). Untreated cells served as reference and untreated cells, also not receiving the induction medium on day 2, as negative control. BrdU incorporation was quantified according to the manufacturer’s instructions (ab126556, Abcam, Cambridge, UK) using a microplate reader (Tecan). VaspinN effects (again: 1 nM and 100 nM, chronically or acutely) on MAPK activation was analysed by Western blot 1 h after adipogenic induction to assess an essential signal transduction pathway in the early phase of MCE. ADIPOQ and PPARG expression was quantified by Western blot 72 h post induction (day 5) after VaspinN peptide treatment. To study VaspinN peptide effects on adipogenic differentiation and lipid incorporation, confluent 3T3-L1 cells (day 0) were treated with VaspinN peptide (1 nM, 10 nM and 100 nM; added to the medium daily). Intracellular lipid accumulation was analysed at day 2 and day 10 after adipogenic differentiation using the AdipoRed reagent (Lonza, Basel, Switzerland) according to the manufacturer’s instructions. Lipid content was quantified measuring fluorescence (572 nm) using a microplate reader (Tecan) or was visualized by fluorescence microscopy. Expression of marker genes was analysed by qPCR.

### SDS-PAGE and Western Blot

Whole-cell protein lysates were extracted with RIPA buffer, subjected to SDS-PAGE, transferred to nitrocellulose membranes using tank blot method and incubated with primary antibodies as previously described [14]. HRP-coupled secondary antibodies were used and chemiluminescence was detected and quantified using the G:BOX Chemi XX9 system with GeneSys and GeneTools software (SynGene, Bengaluru, Karnataka, India). The following antibodies were used: b-Actin (A1978, Thermo Fisher Scientific), phospho-AKT (Ser473) (#4060, Cell Signalling Technologies, Danvers, MA, USA), AKT (#4691, Cell Signalling Technologies), phospho-PKA substrate (RRXS*/T*) (#9624, Cell Signalling Technologies (CST)), p44/42 MAPK (Erk1/2) (#4695, CST), phospho-p44/42 MAPK (Erk1/2; Thr202/Tyr204) (#4370, CST), ADIPOQ (#2789, CST), PPARG (#2430, CST), anti-rabbit-HRP (#7074, CST) and anti-mouse-HRP (#7076, CST).

### In vitro glucose uptake and lipolysis assays

To measure basal and insulin-induced glucose uptake, mature 3T3-L1 cells were incubated in serum-free DMEM in the presence of peptides (0 nM, 1 nM, and 10 nM) for 16 h and low-glucose DMEM 1 h prior to experiment. Following 15 min of insulin stimulation (100 nM), cells were incubated with 3 H-Deoxy-D-glucose (NET549A001MC, Perkin Elmer, Waltham, MA, USA) at an activity of 18.5 kBq/ml for 30 min, lysed with RIPA buffer and lysates were transferred to scintillation vials. Activity was measured using a beta-counter (Perkin Elmer). Total cellular protein concentration was determined using a Pierce BCA Protein assay (Thermo Fisher Scientific) and used for data normalization.

Lipolysis was measured in fully differentiated 3T3-L1 cells incubated with increasing concentrations of peptide (0 nM, 1 nM, 10 nM and 100 nM) for 24 h. Lipolysis was induced by addition of isoprotenerol (final concentration of 100 nM) for 3 h and glycerol release was measured using lipolysis assay kit (ab185433, Abcam) according to the manufacturer’s protocol.

### Statistical analyses

Data are presented as means ± SEM. Statistical analyses were performed using GraphPad Prism7 (GraphPad, San Diego, Ca, USA). Statistical significance was determined by Student’s two-tailed *t*-test, one-way or two-way ANOVA followed by adequate post-hoc tests when comparing multiple groups. *p-*values <0.05 were considered significant.

## Results

### N-terminal peptides of human vaspin are cell-penetrating peptides

**Figure 1.**
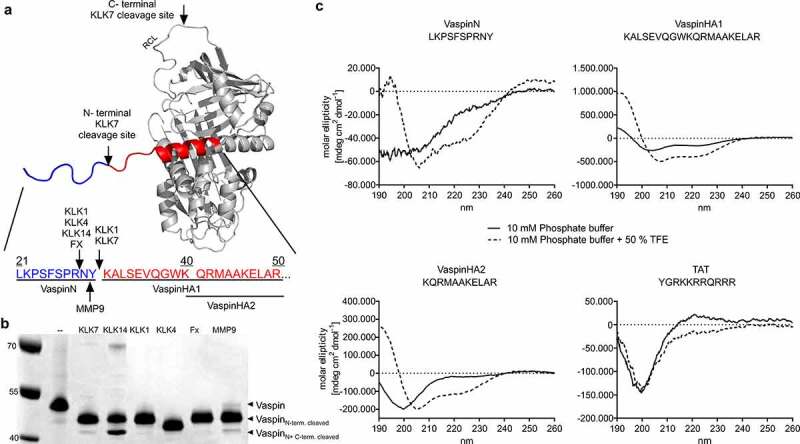
Origin of N-terminal vaspin peptides investigated in this study. a) structural model of vaspin (based on PDB entry 4IF8), and N-terminal cleavage by various proteases. Cleavage sites are indicated by arrows and the endogenous amino acid sequence without the signal peptide (beginning with Leu^21^) is shown. The N-terminal peptide cleaved by target protease KLK7 (VaspinN, blue) and two further N-terminal peptides (VaspinHA1 and VaspinHA2, red) were analysed in this study. b) SDS-PAGE analysis of vaspin processing. Vaspin was incubated with various proteases (serpin:protease molar ratio of 6:1) for 16 h. Indicated bands are full length, N-, as well as N- and C-terminally cleaved vaspin. c) Circular dichroism spectra of vaspin and TAT peptides acquired using 50 µM peptide. Fx: Factor Xa, KLK: Kallikrein, MMP9: matrix metalloproteinase 9, TFE: Trifluoroethanol

We have previously observed specific cleavage in the vaspin N-terminus by both kallikrein target proteases, KLK7 and KLK14, in addition to cleavage within the RCL [16]. KLK7 cleavage after Tyr^30^ releases an N-terminal peptide of 10 amino acids and on the other side generates a truncated vaspin N-terminus, while KLK14 cleaves after Arg^28^ (Figure 1a). Also, multiple other proteases tested for inhibition by vaspin showed cleavage within the vaspin N-terminus to generate N-terminal peptides of similar length, albeit not being inhibited by the serpin (Figure 1b). For example, KLK1 cleaves after vaspin residues Arg^28^ and Tyr^30^, factor X and KLK4 after Arg^28^ and MMP9 after Asn^29^ (Table 1).

**Table 1.** N-terminal vaspin sequences generated by protease cleavage and prediction of cell-penetrating activity using CPPred-RF [24]

Vaspin residues	Sequence	Cleaving Proteases	Cell-Penetrating or not	Prediction Confidence	Uptake Efficiency	Prediction Confidence
Vaspin^21−41^	LKPSFSPRNYKALSEVQG		Non-cell-penetrating	0.560	-	-
Vaspin^21−50^	LKPSFSPRNYKALSEVQGWKQRMAAKELAR		Non-cell-penetrating	0.560	-	-
Vaspin^21−30*^	LKPSFSPRNY -	KLK1, KLK7^§^	Cell-penetrating	0.520	High	0.608
Vaspin^21−29^	LKPSFSPRN -	MMP9	Cell-penetrating	0.560	High	0.600
Vaspin^21−28^	LKPSFSPR -	KLK1, KLK4, KLK14^§^, FX	Cell-penetrating	0.750	High	0.740
Vaspin^31−50*^	– KALSEVQGWKQRMAAKELAR		Cell-penetrating	0.530	High	0.685
Vaspin^40−50*^	– KQRMAAKELAR		Cell-penetrating	0.730	High	0.610

*Peptides synthesized and investigated in this study; Vaspin^21−30^ = VaspinN; Vaspin^31−50^ = VaspinHA; Vaspin^40−50^ = VaspinHA2. ^§^ Target proteases of vaspin [9,12]

We first performed sequence analysis to evaluate potential cell-penetrating activity of N-terminal vaspin peptides, both the released N-terminal peptides and the N-terminal sequence remaining on the serpin molecule. All were predicted to be efficient CPPs (Table 1), with the exception of the sequence comprising the native and uncleaved N-terminus.

We then synthesized fluorescent TAMRA-labelled peptides for fluorescence microscopy studies, resembling the cleaved peptide (vaspin^21−30^, VaspinN) and two peptides comprising the remaining N-terminal sequence of the A+ helix of vaspin (vaspin^31−50^ termed VaspinHA1, and vaspin^40−50^ termed VaspinHA2; Figure 1a). For comparison, we used TAMRA-labelled TAT peptide derived from the transactivator of transcription (TAT) of human immunodeficiency virus [39]. We first analysed peptide structures via CD-spectroscopy (Figure 1c). In line with the vaspin crystal structure [9] and CD-spectroscopy data of the full-length serpin [16], VaspinHA1 and VaspinHA2 peptides exhibit extensive α-helical structure. VaspinN, comprising the first N-terminal amino acids that are not resolved in the crystal structure, did not readily adopt a specific fold, but exhibited propensity to form an α-helical structure in the presence of TFE. CD spectra of the TAT peptide showed a random coil structure (Figure 1c), as also observed by others [40,41].

**Figure 2.**
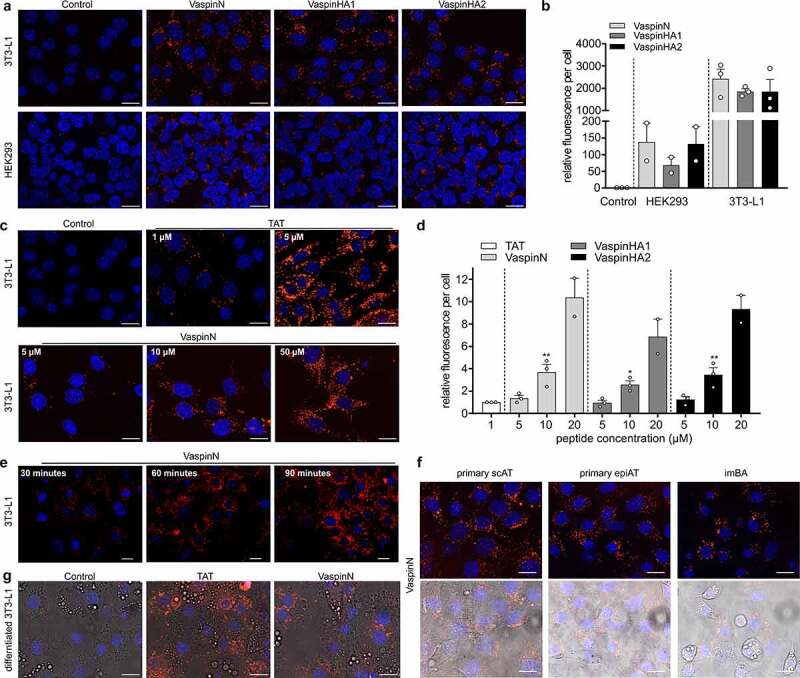
Vaspin peptide internalization in different cell lines. a) Fluorescence microscopy images of control or vaspin peptide treated 3T3-L1 and HEK393 cells (10 µM of respective peptide for 30 min). b) High content imaging-based quantification of relative fluorescence in 3T3-L1 and HEK293 treated as in (A). Data were normalized to control cell fluorescence. c) Fluorescence microscopy images of 3T3–L1 cells incubated with increasing concentrations of TAT and VaspinN peptide (range 1–5 µM and 5-50 µM, respectively). d) Quantification of relative fluorescence in 3T3–L1 cells treated with increasing concentrations (5-20 µM for 30 min) of all three vaspin peptides. Cells treated with 1 µM TAT served as reference. e) Fluorescence microscopy images of 3T3-L1 cells investigating time-dependent internalization of 10 µM VaspinN after 30 min, 60 min and 90 min. f) Fluorescence microscopy images of VaspinN treated primary subcutaneous (scAT), epididymal (epiAT) and immortalized brown adipocytes (imBA) (10 µM of VaspinN for 30 min). g) Fluorescence microscopy images of internalized TAT (1 µM) or VaspinN (10 µM) in differentiated 3T3-L1 adipocytes after 30 min. Scale bar for fluorescence microscopy images: 20 µm. Blue: Hoechst 33342 nuclear stain, red: TAMRA-fluorescence of labelled peptides. Data are presented as mean ± SEM of at least two independent experiments in triplicates

Using fluorescence microscopy, we first examined cellular fate of all peptides in 3T3-L1 adipocytes and HEK293 cells. As predicted, all peptides showed rapid and efficient cell-penetration into 3T3-L1 and HEK293 and HepG2 (Figure 2a, Supplementary Figure S1A for HepG2) and peptide uptake was detectable at concentrations as low as 500 nM (Supplementary Figure S1B). Internalization into 3T3-L1 was more efficient and high content imaging confirmed uptake of vaspin-derived peptides by 3T3-L1 cells to be >10-fold higher than in HEK293 cells (Figure 2b). For all peptides, internalization was followed by accumulation in intracellular vesicles without entering the nucleus. We did not observe nuclear localization of TAT in 3T3-L1 cells (Figure 2c), contrary to other work [42]. Fluorescence microscopy revealed a dose- (Figure 2c for 3T3-L1; Supplementary Figure S1C for HEK293) and time-dependent internalization process (Figure 2e). To achieve uptake and fluorescence in 3T3-L1 cells comparable to the TAT peptide, fivefold higher concentrations of the vaspin peptides were necessary, indicating effective cell penetration (Figure 2d). Finally, we investigated VaspinN internalization in other adipocyte cell lines, including primary subcutaneous and epididymal adipocytes (scAT and epiAT, respectively), immortalized brown adipocytes (imBA), and mature and fully differentiated 3T3–L1 adipocytes (Figure 2f, 2g). All showed rapid and efficient uptake of the VaspinN peptide.

### VaspinN peptide is internalized by an active mechanism

**Figure 3.**
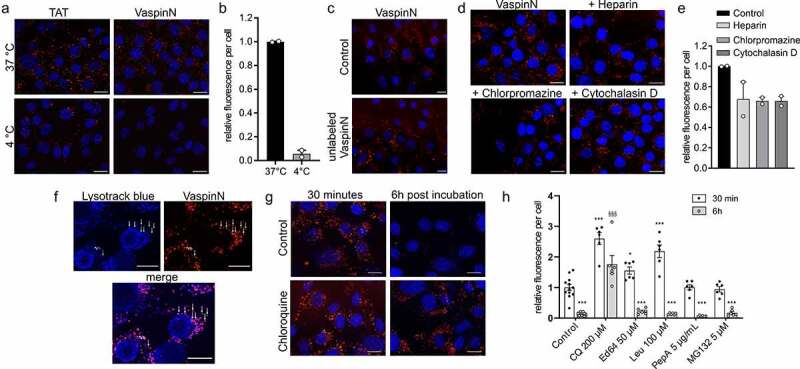
Mechanisms contributing to VaspinN internalization into 3T3-L1 cells. a) Fluorescence microscopy images of 3T3-L1 cells treated with 1 µM TAT and 10 µM VaspinN at 37°C or 4°C for 30 min. b) High content imaging-based quantification of fluorescence in 3T3-L1 cells treated as in (a). c) Preincubation of 3T3-L1 cells with 100 µM unlabelled VaspinN, followed by 10 µM VaspinN for 30 min. d) Fluorescence microscopy images of 3T3-L1 cells after incubation with 10 µM VaspinN for 30 min in the presence of various endocytosis inhibitors. e) High content imaging-based quantification of relative fluorescence in 3T3-L1 cells pretreated with endocytosis inhibitors as in (d). f) Fluorescence microscopy images of 3T3-L1 cells after preincubation with 100 µM chloroquine for 30 min, followed by addition of 10 µM VaspinN. Lysosomes were stained with Lysotracker blue. g) Fluorescence microscopy images of 3T3-L1 cells after preincubation with 100 µM chloroquine for 30 min, followed by addition of 10 µM VaspinN. Images were taken 30 min and 6 h post addition of VaspinN. h) High content imaging-based quantification of relative fluorescence in 3T3-L1 cells pretreated with different protease or proteasome inhibitors followed by 10 µM VaspinN for 30 min. Fluorescence was quantified immediately or 6 h after VaspinN treatment for 30 min. scale bar: 20 µm. Blue: Hoechst 33342 nuclear stain, red: TAMRA-fluorescence of labelled peptides. Data presented as mean ± SEM. Statistical significance was evaluated by two-way ANOVA with Dunnet’s post-hoc test (h) */§ *p*-value <0.05, ***/§§§ *p*-value <0.001; * vs. 30 min control, § vs 6 h control

In the following, we investigated the mechanism of peptide internalization only for VaspinN comprising the cleaved vaspin amino acids 21-30, as this is released by protease cleavage. Reduction of ambient temperature (to 4°C) significantly decreased internalization into 3T3-L1 for VaspinN (Figure 3a, 3b) and also for TAT, as previously described [43]. These data indicate that an active energy-dependent mechanism is underlying peptide uptake. Competition with high concentrations of unlabelled peptide (100 µM) did not affect peptide uptake indicating that translocation of the peptide is independent of a specific cell surface receptor (Figure 3c). We then analysed and quantified internalization in the presence of inhibitors of clathrin-mediated endocytosis and micropinocytosis (Figure 3d). We also considered an excess of heparin, to investigate whether interactions with glycosaminoglycans are involved in peptide uptake. We observed marked reductions in internalization of ~30% for all conditions tested, indicating that multiple mechanisms including clathrin-mediated endocytosis and micropinocytosis pathways are involved in peptide uptake (Figure 3e). The peptide then accumulates in lysosomes, as indicated by the superposition of VaspinN TAMRA-fluorescence with lysotracker blue (Figure 3f), and is finally degraded after 6 h (Figure 3g). We then used various concentrations of leupeptin, Ed64 and pepstatin A to investigate contributions of lysosomal serine, cysteine and aspartyl proteases to peptide degradation, as well as MG132 to delineate degradation by the proteasome in the cytosol after potential release/escape from the endosome (Supplementary Figure S2). Only chloroquine increased CPP fluorescence signal both directly after peptide incubation (30 min) and 6 h later (Figure 3g). Of the protease inhibitors used, leupeptin and Ed64 treatment resulted in significantly increased fluorescence after 30 min of VaspinN incubation, but none of the inhibitors did prevent peptide degradation over 6 h and CPP fluorescence was reduced to levels of untreated controls (~10-15% of initial fluorescence) (Figure 3h). These experiments indicate endosome acidification as the primary determinant of VaspinN stability, and both leupeptin and Ed64 reduced proteolytic degradation, but not to the extent of chloroquine.

### The VaspinN peptide shows no cell toxicity

To determine potential toxicity of VaspinN in 3T3-L1 cells, we performed lactate dehydrogenase (LDH) assays after incubating cells for 24 h with VaspinN at different concentrations of up to 100 µM. We did not see any effect on cell morphology (data not shown) nor in LDH-activity in cell supernatants (Supplementary Figure S3).

### Specific changes in gene expression of primary adipocytes indicate vaspin peptide effects on adipocyte proliferation, differentiation and lipolysis

**Figure 4.**
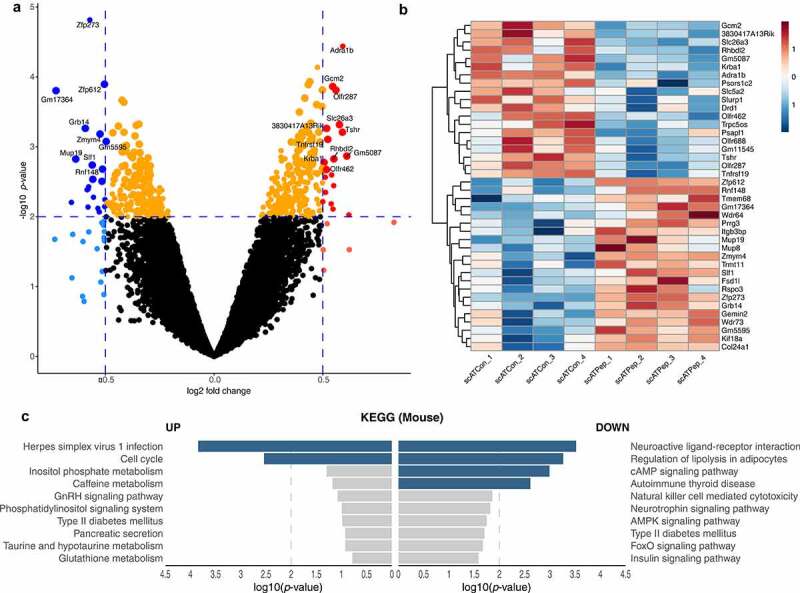
Microarray analysis of gene expression changes induced by VaspinN peptide in primary adipocytes. a) Volcano plot of DEGs in VaspinN-treated (scATPep) vs. control (scATCon) primary subcutaneous adipocytes. Microarray gene expression data in the volcano plot are displayed as log_2_ fold change (FC) versus the -log_10_ of the *p-*value. downregulated genes in scATPep (-log_10_ *p-*value >2 and log_2_ FC ≤ −0.5) compared to scATCon are shown in blue, whereas red colour codes upregulated genes in scATPep (-log_10_ *p-*value >2 and log_2_ FC ≥ 0.5). Dots in orange indicate a *p*-value <0.001. Thresholds are shown as dashed lines. The top 20 DEGs (sorted by log-odds) are labelled with gene symbols. b) Heatmap with scATPep vs. scATCon cells with *p*-value <0.01 and |log_2_ FC| ≥0.5. Red indicates genes higher expressed in scATPep compared to scATCon, whereas blue represents lower expressed genes. c) KEGG pathway functional enrichment analysis of DEGs (top ten functional pathways up- and downregulated). The vertical axis represents the KEGG pathway terms significantly enriched by the DEGs; the horizontal axis indicates -log_10_ (*p*-value)

We then performed microarray analysis of gene expression changes in fully differentiated mouse primary subcutaneous adipocytes after 24 h incubation with VaspinN (100 nM) to evaluate potential effects of the CPP on adipocyte biology and function. In total, N = 40 genes were differentially expressed in VaspinN treated cells compared to controls (*p*-value <0.01, |log_2_ FC| ≥0.5; Figure 4a; Supplementary Table S3). N = 21 genes were upregulated and N = 19 genes showed decreased expression after CPP treatment (Figure 4b). To explore the biological functions of the identified DEGs pathway (KEGG) and functional annotation (GO term) analyses were performed using Enrichr. Gene list analyses (using all DEGs with a *p*-value <0.01; N = 467, including 239 up- and 228 downregulated genes) revealed significant enrichment of genes related to adipocyte biology. KEGG pathway analyses revealed that gene expression was significantly increased for genes related to cell cycle (such as *Orc2, Orc4, Orc5, Cdc20* and *Chek1*; Figure 4c, Supplementary Table S4). Significant enrichment in downregulated genes was related to neuroactive ligand–receptor interaction, the regulation of lipolysis in adipocytes and cAMP signalling pathways (such as *Lipe, Adora1, Pik3cd, Irs2* and *Tshr*; Supplementary Table S5). GO analysis (Supplementary Figure S4) revealed enrichment of genes associated with DNA replication (initiation, GO:0006270 and DNA-dependent, GO:0006261) in upregulated genes (Supplementary Table S6). On the other hand, genes involved in adenylate cyclase-activating G-protein coupled receptor, calcium- and cAMP-mediated signalling (GO:0007189, GO:0019722, GO:0007188, GO:0019933) and response to glucose (GO:0009749) were enriched in downregulated genes (Supplementary Table S7).

### The N-terminal vaspin peptide has specific effects on preadipocyte proliferation, mitotic clonal expansion and mature adipocyte metabolism

**Figure 5.**
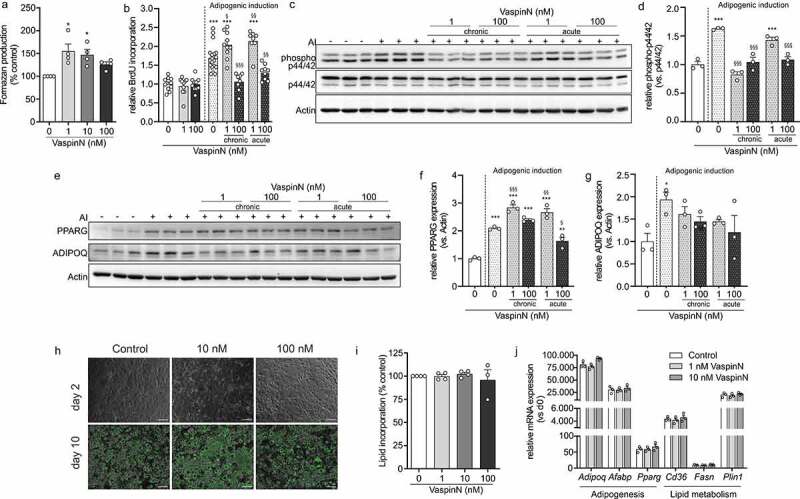
Biological effects of VaspinN peptide– preadipocyte metabolic activity, mitotic clonal expansion and adipogenesis. a) 3T3-L1 cells were cultured for 24 h in the presence of increasing concentrations of VaspinN (0 – 100 nM) and formation of formazan dye was quantified. b) BrdU incorporation into 3T3-L1 preadipocytes 24 h after adipogenic induction (AI) was differentially affected by VaspinN at 1 nM and 100 nM. c, d) Western Blot analysis of p44/42 MAPK phosphorylation 1 h after adipogenic induction (c) in post-confluent 3T3-L1 preadipocytes treated with or without 1 nM or 100 nM of VaspinN (acutely and chronically). Panels: anti-phospho-p44/42 MAPK antibody, p44/42 MAPK antibody and Actin antibody. Chemiluminescence was quantified and normalized to controls (d). VaspinN significantly inhibited at 100 nM significantly inhibited p44/42 MAPK phosphorylation. e-g) Western Blot analysis of PPARG and ADIPOQ expression three days after adipogenic induction (e) in 3T3-L1 cells treated with or without 1 nM or 100 nM of VaspinN (acutely and chronically). Panels: PPARG antibody; ADIPOQ antibody and Actin antibody. Chemiluminescence was quantified and normalized to controls (f, g). VaspinN only inhibited expression of PPARG and ADIPOQ at 100 nM. h) Fluorescence microscopy of control and VaspinN-treated (10 nM, 100 nM) fully differentiated 3T3-L1 adipocytes. Lipid droplets were stained using AdipoRed. Pictures at day two and day ten are representative of three independent experiments. i) Quantiﬁcation of lipid accumulation revealed no significant changes in adipogenesis upon differentiation (day 10) in the presence of VaspinN peptide. j) Expression of marker genes of adipogenesis (*Adipoq, Afabp, Pparg*) and lipid metabolism (*Cd36, Fasn, Plin*) increased during differentiation with no differences between treatments. Data are shown as mean ± SEM. Statistical significance was evaluated by one-way ANOVA with Sidak post-hoc test (a, b, d, f, g). */§ *p*-value <0.05, **/§§ *p*-value <0.01, ***/§§§ *p*-value <0.001; * vs. control, § vs adipogenic induction (AI)

Following up on the microarray analysis, we performed experiments investigating effects of VaspinN on 3T3-L1 preadipocyte proliferation and differentiation, as well as on metabolic activity of differentiated 3T3-L1 adipocytes. After incubation for 24 h with increasing concentrations of VaspinN (1 nM, 10 nM and 100 nM), measurements of formazan dye formation as a consequence of WST-1 tetrazolium salt cleavage indicated significantly higher cellular metabolic activity of 3T3-L1 preadipocytes in response to 1 nM and 10 nM of VaspinN (Figure 5a).

We next investigated potential effects of the VaspinN peptide on clonal expansion of 3T3-L1 cells by analysing DNA synthesis, activation of MAPK signalling and protein expression of PPARG and ADIPOQ after adipogenic induction. We used bromodeoxyuridine (BrdU) to quantify DNA synthesis 24 h post induction of adipogenic differentiation after cells had been continuously or acutely treated with VaspinN peptide (1 nM and 100 nM) (Figure 5b). We observed significant and dose-dependent effects on clonal expansion, with a ~ 10% increase in BrdU incorporation in cells treated with 1 nM VaspinN and significant inhibition of clonal expansion in cells treated with 100 nM VaspinN. We further analysed early p44/42 MAPK phosphorylation (1 h post induction; Figure 5c, 5d), PPARG and ADIPOQ protein expression (72 h post induction; Figure 5e-g). Chronic treatment with 1 nM VaspinN significantly suppressed p44/42 MAPK phosphorylation, while there were no acute effects. During post-mitotic growth arrest, 1 nM VaspinN did significantly increase PPARG expression, but had no effect on ADIPOQ expression. In line with the results from the BrdU assays, all 3T3-L1 cells treated with 100 nM VaspinN showed significantly reduced p44/42 MAPK phosphorylation, which in part caused a reduction of PPARG and ADIPOQ expression 72 h after adipogenic induction, though these differences did not reach statistical significance. Ultimately though, 3T3-L1 cells did fully differentiate in the presence of 1 nM, 10 nM and 100 nM VaspinN (Figure 5h). Quantification of lipid incorporation and fluorescence microscopy using the lipophilic AdipoRed reagent did not reveal any differences after terminal differentiation compared to untreated control cells (Figure 5i). In line, expression of key adipogenic marker genes reflected full differentiation of 3T3-L1 adipocytes which were unaffected by peptide treatment (Figure 5j).

**Figure 6.**
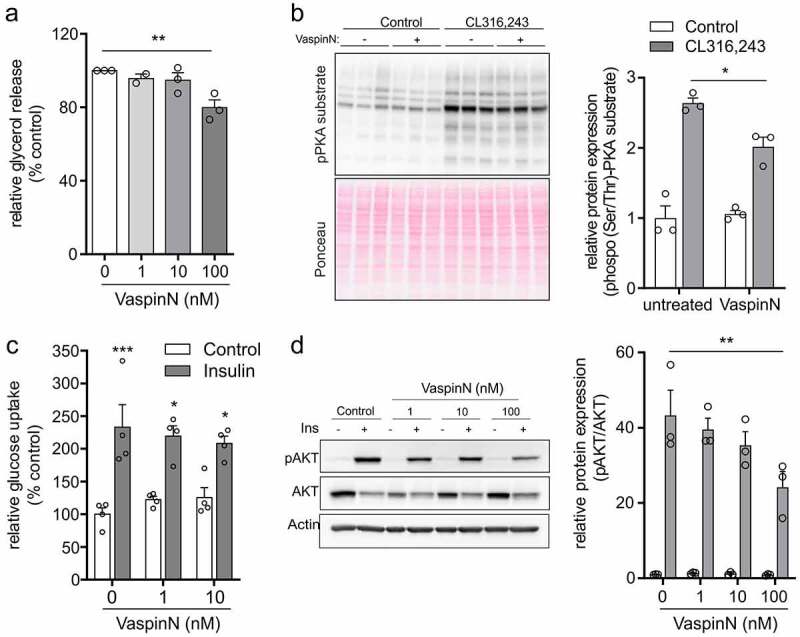
Biological effects of VaspinN peptide – lipolysis and glucose metabolism. a) Measurement of glycerol in supernatants of differentiated 3T3-L1 adipocytes in response to 100 nM isoprotenerol after 24 h incubation with increasing concentrations of VaspinN (0 – 100 nM). VaspinN dose-dependently blunted lipolysis and resulting glycerol release. b) Western Blot analysis of PKA activation by β-adrenergic agonist CL316,243 in differentiated 3T3-L1 adipocytes after treatment with or without 100 nM of VaspinN for 24 h. upper panel: anti-phospho-PKA substrate antibody; lower panel: loading control using Ponceau. Chemiluminescence was quantified and normalized to controls. VaspinN significantly inhibited PKA activation. c) Insulin-induced glucose uptake into differentiated control and VaspinN pretreated (1 nM, 10 nM for 24 h) 3T3-L1 adipocytes (N = 4). d) Insulin-induced AKT phosphorylation analysed by Western Blot and quantified by ECL. Differentiated 3T3-L1 adipocytes were treated with or without 1 nM, 10 nM and 100 nM of VaspinN for 24 h, followed by insulin (100 nM for 15 min). VaspinN did not affect insulin-mediated glucose uptake and but impaired AKT phosphorylation. Data are shown as mean ± SEM of at least two independent experiments in duplicates or triplicates. Statistical significance was evaluated by one-way or two-way ANOVA with Tukey post-hoc test. *p-value <0.05, **p-value <0.01

To address VaspinN effects on adipocyte lipolysis and cAMP signalling pathways, that were indicated by microarray analysis, we investigated cAMP signalling in differentiated 3T3-L1 adipocytes in response to selective β3-adrenergic agonist CL316,243 in control and VaspinN-treated (1 nM, 10 nM and 100 nM) cells. First, VaspinN significantly decreased glycerol release in differentiated 3T3-L1 adipocytes in a dose-dependent manner (Figure 6a). Also, phosphorylation of PKA substrates in response to selective β3-adrenergic receptor agonist CL316,243 (Figure 6b) or adenylyl cyclase activator forskolin (Supplementary Figure S5) was blunted. Finally, we also measured in vitro glucose uptake as well as intracellular insulin signalling in VaspinN-treated differentiated 3T3-L1 adipocytes in response to acute insulin stimulation (Figure 6c, 6d). Here, insulin-induced glucose uptake was not different for the VaspinN concentrations used, but AKT phosphorylation was reduced in cells treated with 100 nM VaspinN.

## Discussion

While testing various proteases for inhibition by the serpin vaspin, we frequently observed specific cleavage of the vaspin N-terminus. Sequence analysis predicted cell-penetrating activity for the released peptides. These observations raised the question whether these proteolytic peptides exhibit biological activity.

Focusing on adipocytes, our study provides first evidence of a novel aspect of vaspin function that is mediated by short and effective amphipathic cell-penetrating peptides derived from the serpins N-terminus. Cell penetration was fast, efficient and readily detectable after 30 min at concentrations as low as 500 nM, but ~fivefold less efficient than the TAT reference. Uptake of the CPP released by KLK7–cleavage (VaspinN, comprising vaspin residues 21-30) into 3T3-L1 adipocytes was more efficient than uptake into HEK293 and HepG2 cells. For HEK293, similar differences in TAT-uptake compared to 3T3-L1 indicate that lower peptide uptake per cell is at least in part due to less cytosolic area per nucleus (data not shown). But HepG2 cells with similar cytosol-to-nucleus ratio to 3T3-L1 (Supplementary Figure S1A) showed significantly less peptide uptake, indicating some degree of cell-specificity or preference.

Internalization of CPPs is achieved via two main mechanisms, which are either energy-dependent endocytosis or direct translocation through the cell membrane [44]. For the vaspin-derived CPP, we show that active endocytosis is mainly underlying cellular uptake and that different pathways are contributing. Inhibition of clathrin-mediated endocytosis as well as caveolar endocytosis and macropinocytosis all partially reduced cellular uptake into adipocytes. The presence of heparin also did reduce endocytosis, indicating that interaction with cell surface proteoglycans is involved as an initial step before internalization. The contribution of multiple uptake mechanisms is not uncommon and has been reported for CPPs derived from N-terminal sequences of human annexin A isoforms [45]. Excess of unlabelled peptide did not affect internalization, precluding importance of binding to a specific cell surface receptor such as the serpin-enzyme complex receptor LRP1 [46].

The vaspin-derived CPP then is trafficked into a vesicular compartment and blocking endosomal acidification and activation of endo/lysosomal proteases increased or prolonged intracellular accumulation by preventing endosome-lysosome fusion and subsequent peptide degradation [47]. In our experiments, inhibition of endo/lysosomal proteases did only increase VaspinN fluorescence 30 min after CPP incubation, but did not prevent VaspinN degradation over the long term. Yet, this is likely due to the fact that cells were only pretreated with the inhibitors and inhibitors were not present during the following 6 h. Finally, inhibition of proteasomal degradation in the cytosol did not affect short- or long-term intracellular peptide fluorescence, indicating that VaspinN does not exhibit endosomolytic activity and does not enter the cytoplasm.

The vaspin-derived peptides showed no cytotoxic effects on adipocytes and microarray data revealed adipocyte-specific changes in gene expression after peptide treatment of fully differentiated primary subcutaneous adipocytes. These changes were linked to proliferation, lipolysis and cAMP and, to lesser extent, insulin signalling. Subsequent functional assays confirmed that the VaspinN peptide indeed increased preadipocyte proliferation, while it did not affect adipocyte adipogenesis and differentiation. In mature adipocytes, VaspinN blunted adrenergic cAMP-signalling, downstream lipolysis as well as insulin signalling. These data demonstrate functional activity of these novel vaspin-derived peptides.

Obesity is the major risk factor for type 2 diabetes mellitus, atherosclerosis and non-alcoholic fatty liver disease [48]. Chronic low-grade inflammation of the adipose tissue as a consequence of adipocyte hypertrophy contributes to the development of insulin resistance [49]. Previous studies have demonstrated vaspin's anti-inflammatory role in adipose tissue. Administration and overexpression of vaspin blunted obesity-induced inflammation in adipose tissue in mice and rats [8,10,11]. The mechanisms by which vaspin exerts these beneficial, protective and/or compensatory effects on adipocyte function have not been fully unravelled, with studies demonstrating both effects via protease-inhibition as well as receptor binding, indicating multifaceted actions of the serpin [8,9]. In adipose tissue, protease inhibition by vaspin is likely a major contributing mechanism to modulate inflammation, as knock out of target protease KLK7 in adipose tissue significantly reduced adipose tissue inflammation and insulin resistance in obese mice [15]. Also obese mice overexpressing vaspin in adipose tissue exhibit adipocyte hyperplasia and reduced adipocyte hypertrophy in visceral adipose tissue [8]. Direct vaspin effects on adipocyte differentiation are controversial. While one study reported a minor positive and dose-dependent effect of vaspin on adipocyte lipid incorporation and adipogenic gene expression in 3T3-L1 cells [50], we did not observe this, neither with exogenous vaspin nor vaspin overexpression [14]. Along these lines, vaspin-derived N-terminal peptides may contribute to increase local proliferation of preadipocytes during adipose tissue expansion, thereby counteracting adipocyte hypertrophy, inflammation and dysfunction and preserving insulin sensitivity in obesity [51].

VaspinN does clearly affect early stages of adipogenesis in preadipocytes. While low concentrations (1 nM) did enhance preadipocyte metabolic activity and mitotic clonal expansion, higher concentrations of VaspinN (100 nM) significantly inhibited MAPK/ERK signalling in the early G1 phase after adipogenic induction and this interference in part persisted and carried over into the post-mitotic arrest phase, resulting in lower PPARG levels and ADIPOQ expression. The primarily insulin-induced activation of preadipocyte AKT and MAPK pathways during the initiation of clonal expansion is required for adipogenesis and terminal differentiation [52]. But VaspinN likely did not fully prevent or block clonal expansion and underlying signalling pathways, at least in the concentrations used in this study. In consequence, adipogenesis and differentiation of VaspinN-treated cells ultimately seem to catch up with untreated controls, and marker gene expression as well as lipid incorporation were indistinguishable ten days after adipogenic induction. This is again in line with previous findings, showing that insufficient inhibition of MAPK activation did only delay clonal expansion, but did not ultimately prevent adipogenesis [38]. Only full blockade of MAPK activation, e.g. using the highly potent MAPK inhibitor U0126, did block clonal expansion and prevented adipogenesis [52]. In addition to the MAPK/ERK pathway, VaspinN may also affect the insulin-PI3K-AKT pathway, as observed in fully differentiated primary adipocytes, and thereby affect cell-cycle progression in 3T3-L1 preadipocytes. We found no indication for release of VaspinN into the cytoplasm and the mechanisms how endocytosed VaspinN may interfere with these pathways remain unclear. Nevertheless, these findings suggest potential to utilize vaspin-derived peptides or derivatives thereof to target adipose tissue expansion in the early stage of adipogenesis.

Exposing fully differentiated and lipid-loaded primary adipocytes from subcutaneous white adipose tissue to VaspinN did significantly blunt adrenergic cAMP signalling. As indicated by reduced expression of various genes related to cAMP signalling in the microarray, VaspinN peptide blunted β-adrenergic activation of PKA and subsequent catabolic breakdown of triglycerides as measured by lower adipocyte glycerol release. Adipocyte dysfunction with increased and uncontrolled release of free fatty acids is a major cause of dyslipidemia in obesity, promoting insulin resistance, atherosclerosis and increasing cardiovascular risk [53]. Vaspin expression in human adipose tissue is induced in obesity, and serum levels of vaspin are increased in obesity and type-2 diabetes [54]. This may be a direct response to increased proteolytic activity especially from serine proteases in serum observed in type-2 diabetic patients and also in adipose tissue of insulin resistant obese mice [55]. As a consequence of higher vaspin expression and increased protease activity in obesity, generation of vaspin-derived CPPs is likely to be increased as well. This then may beneficially affect adipose tissue expansion by increasing preadipocyte proliferation, and by limiting lipid flux from hypertrophic adipocytes, thus counteracting dyslipidemia and ectopic fat deposition and insulin resistance, for example, in the liver.

Thus, the generation of functional proteolytic peptides may present a second mechanism by which vaspin-protease interactions modulate obesity-induced inflammatory processes in adipose tissue in addition to the known inhibition of pro-inflammatory proteases [15,55]. We have previously found, that vaspin expression is induced in brown adipose tissue after thermogenic activation [56]. Thus, the inhibitory effect of VaspinN peptides on cAMP signalling and lipolysis may be of specific relevance in regulating BAT thermogenesis and may indicate autoregulatory function of vaspin in this tissue.

One obvious limitation of this study is that it remains unclear, whether these peptides are generated in vivo. Processing of the vaspin N-terminus by multiple proteases will likely result in tissue- and/or disease-specific appearance of diverse vaspin-derived peptides (Figure 1a) and also respective proteases involved remain unknown. Methods to detect and quantify vaspin-derived peptides in blood or tissues are necessary to address these open questions and the aim of ongoing research.

Beyond that, many CPPs have been used to deliver a variety of molecules such as peptides, radionuclides, siRNA or drugs into various cell lines and types [57]. Whether the vaspin-derived CPP reported in this study are capable of efficient cargo-transport into cells and whether it is possible to utilize them for an adipocyte-specific or targeted delivery remains to be investigated.

Together, our study provides evidence that proteolytic processing of the vaspin N-terminus releases cell penetrating and bioactive peptides with effects on adipocyte biology. Future studies are warranted to gain detailed mechanistic insight into these biological activities – in adipocytes, but also in other vaspin target tissues such as liver, skin or the vasculature– and these may clarify, if synthetic vaspin-derived peptides may have potential as therapeutic agents for the treatment of metabolic diseases.

## Supplementary Material

Supplemental Material
