# Supplementary material for: Cleavage of the vaspin N-terminus releases cell-penetrating peptides that affect early stages of adipogenesis and inhibit lipolysis in mature adipocytes
Source: Adipocyte. 2021 Apr 19;10(1):216–31. doi: 10.1080/21623945.2021.1910154 (PMC8078822; doi:10.1080/21623945.2021.1910154)
Supplement: Supplemental Material [file KADI_A_1910154_SM6884.zip › KADI-2020-0093 supplementary material.pdf]

Cleavage of the vaspin N-terminus releases cell-penetrating peptides that affect early stages of adipogenesis and inhibit lipolysis in mature adipocytes

Catherine A. Tindall, Estelle Erkner, Jan Stichel, Annette G. Beck-Sickinger, Anne Hoffmann, Juliane Weiner, John T. Heiker

Supplementary Figures

Supplementary Figure 1

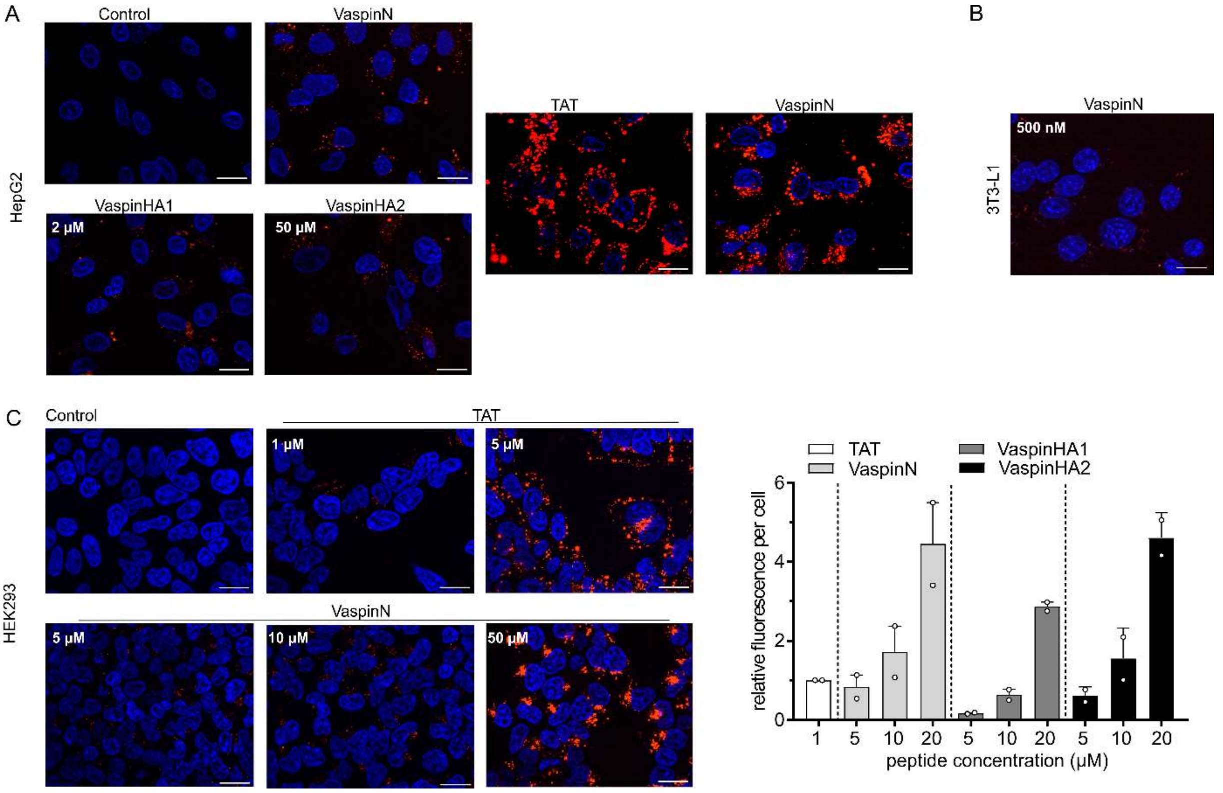

Supplementary Figure 1: A) Vaspin peptide internalization into HepG2 cells. Upper panel: Fluorescence microscopy images of control or vaspin peptide treated cells (20 µM of respective peptide for 30 min). Lower panel: Fluorescence microscopy images of cells treated with TAT (2 µM) or VaspinN (50 µM) for 30 min. B) Fluorescence microscopy images of VaspinN internalization into 3T3-L1 cells at 500 nM. C) Fluorescence microscopy images of cells incubated with increasing concentrations of TAT and VaspinN peptide (range 0-5 µM and 5-50 µM for 30 min, respectively) and quantification of relative fluorescence in cells treated with increasing concentrations (5-20 µM for 30 min) of all three vaspin peptides. Cells treated with 1 µM TAT served as reference. Scale bar for fluorescence microscopy images: 20 µM. Blue: Hoechst 33342 nuclear stain, red: TAMRA-fluorescence of labelled peptides. Data were normalized to the fluorescence of reference cells and are presented as mean ± SEM of at least 2 experiments in triplicates.

Supplementary Figure 2

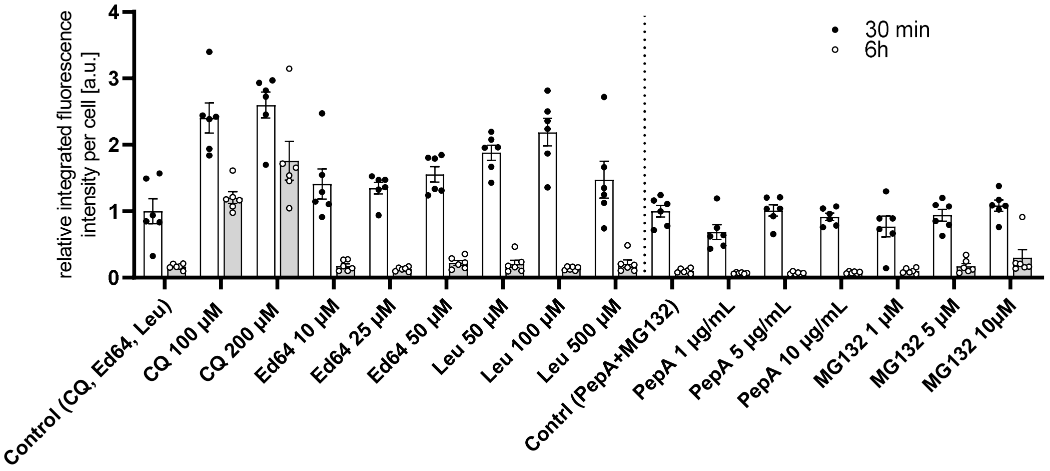

Supplementary Figure 2: High content imaging-based quantification of relative fluorescence in 3T3-L1 cells pretreated with various concentrations of different protease and proteasome inhibitors followed by 10 µM VaspinN for 30 min. Fluorescence was quantified immediately or 6 h after 30 min VaspinN treatment. Data are presented as mean ± SEM.

Supplementary Figure 3

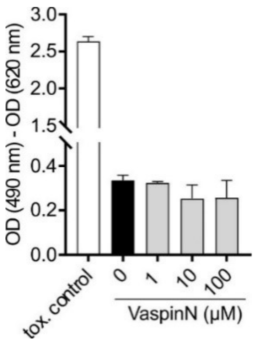

Supplementary Figure 3: LDH Assay of 3T3-L1 cells. OD_{490nm} values of cells treated with different vaspinN concentrations (0-100 µM) for 24h. Control cells were treated with 1% Triton X-100. Data are presented as mean ± SEM of at least 3 experiments.

Supplementary Figure 4

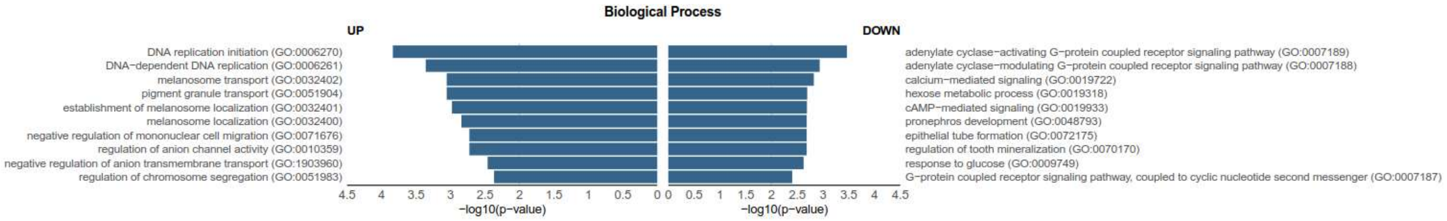

Supplementary Figure 4: Enriched GO terms related to biological processes in DEGs (up and down regulated) after VaspinN treatment in differentiated primary subcutaneous adipocytes.

Supplementary Figure 5

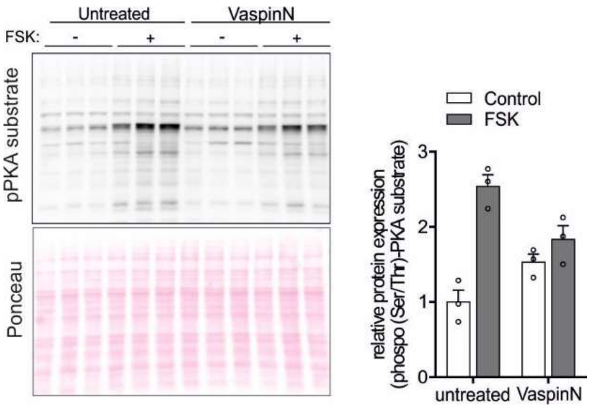

Supplementary Figure 5: Western Blot analysis of PKA activation by adenylyl cyclase activator forskolin in differentiated 3T3-L1 adipocytes after treatment with or without 100 nM of VaspinN for 24 h. Upper panel: anti-phospho-PKA substrate antibody; lower panel: loading control using Ponceau S. Chemiluminescence was quantified and normalized to untreated controls. VaspinN inhibited PKA activation.
